# Fibrous Biomaterial Scaffold for Tympanic Membrane Repair: Microarchitectural Engineering and Structure Function Performance

**DOI:** 10.3390/jfb17010053

**Published:** 2026-01-21

**Authors:** Lea Jiang, Chokri Cherif, Michael Wöltje

**Affiliations:** Institute of Textile Machinery and High Performance Material Technology, TUD Dresden University of Technology, 01069 Dresden, Germany; lea.jiang@tu-dresden.de (L.J.);

**Keywords:** otology, tympanic membrane, tympanic membrane perforation, hearing loss, microarchitecture, biomaterials, electrospinning, additive manufacturing, scaffold design, fibrous biomaterial scaffolds

## Abstract

Tympanic membrane (TM) perforations, arising from infections, injuries, or chronic otitis media, remain a frequent clinical finding and can lead to hearing problems when the tissue does not regenerate adequately. Although autologous grafts are still the standard option for repairing persistent defects, they come with well-known limitations. Beyond the need for additional harvesting procedures, these grafts rarely reproduce the intricate, fibrous layering of the native TM, which can compromise sound transmission after healing. In search of alternatives, fibre-based scaffolds have attracted considerable interest. The primary advantage of this material is the level of structural control it affords. The fibre orientation, porosity, and overall microarchitecture can be adjusted to replicate the organisation and mechanical behaviour of the natural membrane. A range of biocompatible polymers—among them silk fibroin, poly(ε-caprolactone), poly(lactic acid), and poly(vinyl alcohol) and their composites—provide options for tuning stiffness, degradation rates, and interactions with cells, making them suitable building blocks for TM repair constructs. This review provides a comprehensive overview of contemporary fabrication methodologies, namely electrospinning, additive manufacturing, melt electrowriting, and hybrid strategies. In addition, it offers a detailed discussion of the evaluation procedures employed for these scaffolds and discusses how scaffold structure affects later performance. Mechanical testing, microstructural imaging, and in vitro biocompatibility assays help to determine how closely a construct can approach the performance of the native tissue. Bringing these elements together may support the gradual translation of fibre-based TM scaffolds into clinical practice.

## 1. Structure and Anatomy of the Tympanic Membrane

**Figure 1:**
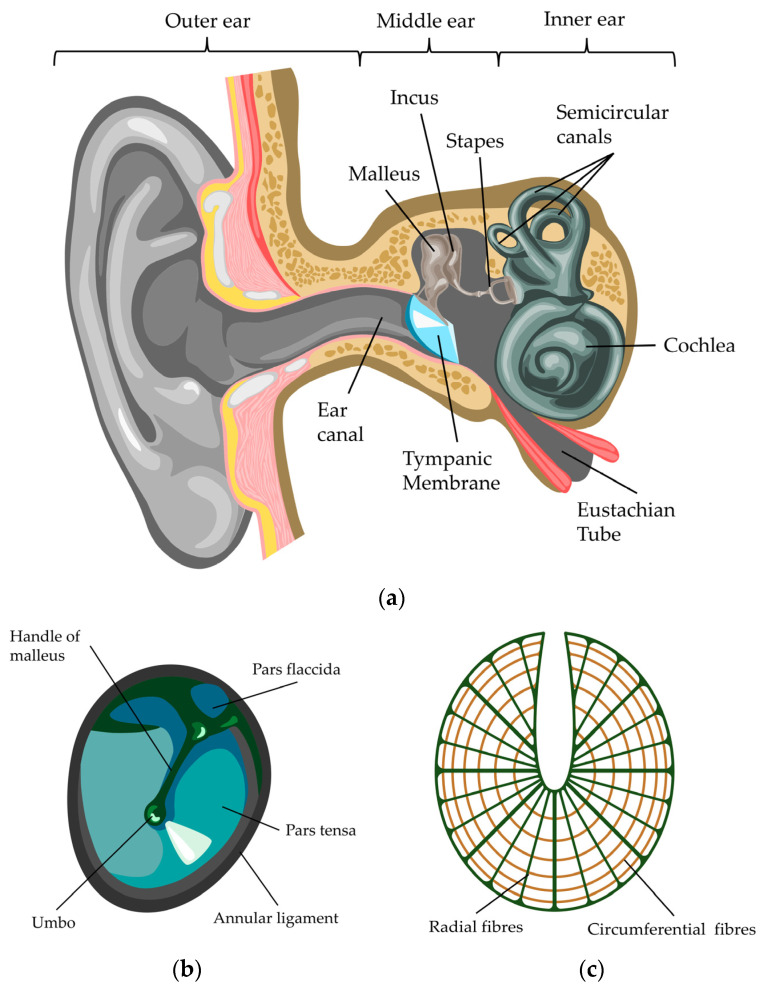
Anatomy of the human ear and the tympanic membrane. (**a**) Frontal section of the human ear, with depiction of the outer ear, ear canal, tympanic membrane, ossicular chain (malleus incus, stapes), cochlea, semicircular canals, cochlea and eustachian tube. (**b**) A more detailed look at the tympanic membrane in top view: detection of handle of malleus, umbo, pars flaccida, pars tensa, and the annular ligament. (**c**) Top view of collagen fibres arranged in the tympanic membrane within the lamina propia; the radial pattern is depicted in green, the circumferential pattern in yellow.

The tympanic membrane (TM), also commonly known as eardrum, is a thin, translucent structure, located at the end of the ear canal, separating the outer ear from the inner ear. To hear, sound is collected by the auricle and then conveyed by the outer ear canal to the TM. Under sound pressure, the TM vibrates and relays the vibrations to the ossicles which transfer it to the cochlea in the inner ear, displacing hair cells and thus triggering the generation of electric signals to be sent to the brain (Figure 1a) [1,2].

A sensitive ear responds to frequencies between 20 to 20,000 Hz. Human ears are most sensitive in the range of 1500 to 4000 Hz, which covers human speech [3,4]. Its resonance frequency, at which the entire membrane vibrates in phase, is approximately 1 kHz [5].

Under physiological conditions the vertical axis of the TM ranges from values between 8.5 to 10 mm, with the horizontal axis ranging between 8 to 9 mm, and an area approximately of 85 mm^2^ [6]. Thus, the shape of the TM can be described as a slightly oval cone, with its apex located centrally at an angle of 132 to 137° and a depth of 1.42 to 2.00 mm, pointing medially [7,8,9,10].

To connect the TM to the ossicular chain, the manubrium of the malleus is coupled to the TM; the connection stretches from the superior edge of the TM, where the lateral process of the malleus is connected, to the umbo, where the connection is tight; however, the connection of the midway region separates slightly from the TM [11]. The TM consists of two regions; the pars tensa (PT) and the pars flaccida (PF) (Figure 1b) [12,13]. The PT occupies the major part of the TM and is a multilaminar structure, while the PF is found in the superior region of the TM. In humans, the PF is a relatively small triangular membrane and differs from the PT in both anatomy and function, since it is thicker and more flaccid [14]. To firmly anchor the TM to the tympanic cavity, the circumferential rim of the PT is thickened as the tympanic annulus and inserts into the tympanic sulcus of the tympanic bone [15].

**Figure 2:**
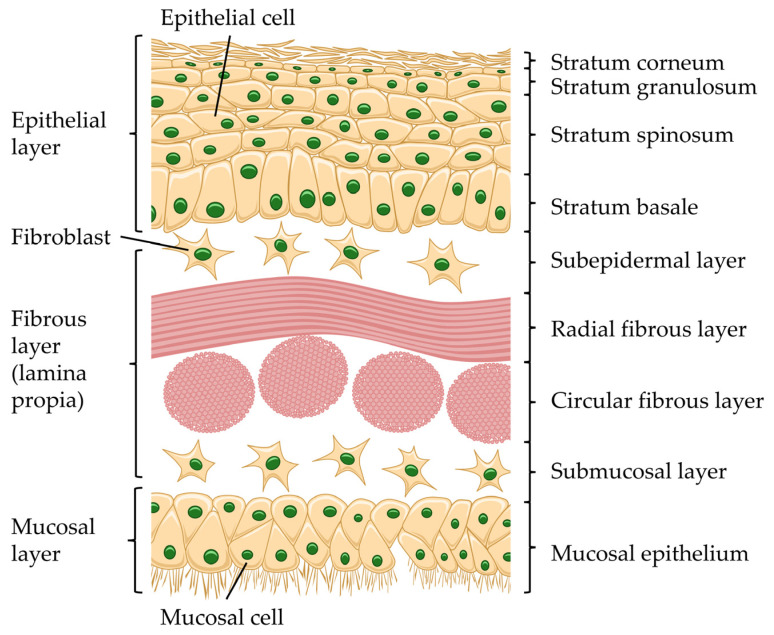
Cross-sectional view of the pars tensa of the human tympanic membrane. The tri-laminar structure of the human tympanic membrane with its three distinct layers (epithelial layer, fibrous layer, and mucosal layer).

In detail, the TM presents as trilaminar structure. The outer layer (lateral side) exhibits stratified squamous epithelium, consisting of keratinocytes. The middle layer is composed of fibroblasts and collagen type II and III; also called lamina propria; herein the collagen provides mechanical strength and elasticity. The inner layer is mucosal and not composed of keratinocytes (Figure 2) [6,16,17,18]. The collagen fibres are arranged in the lamina propria of the PT in two distinct layers; the outer layer exhibits predominantly type II collagen and a lesser extent of type III and I collagen; here, the collagen fibres are arranged radially. The circular collagen fibres of the inner layer of the lamina propria are mainly composed of type III collagen (Figure 1c) [19,20]. The PF is also composed of three layers, but in contrast to the highly ordered collagen fibre structure in the PT, the collagen and elastic fibres in the PF are arranged irregularly, with the addition of blood vessels and nerve endings [12,21]. The inner mucosal layer is continuous with the lining of the tympanic cavity [16].

The acousto-mechanical properties of a physiological TM are highly influenced by its intrinsic structure of collagen fibres, as well as additional elastin and its inherent shape [6,22]. The according hardness varies across different parts of a physiological TM; as collagen fibres are abundant throughout the extracellular matrix (ECM) of the TM, the mechanical hardness mainly depends on them [23]. Young’s modulus is reported to be between 20 to 60 MPa [24,25].

The thickness of the TM is non-uniform and varies across its different parts [26]. The thinnest part of the TM are the central parts of the PT, thickness increases, however, towards the tympanic annulus and umbo [27]. The thickest part of the TM is the PF with a thickness of 78.3 to 373.6 µm and significantly thicker than the PT. The greatest thickness of the PT was found to be in the posterior superior quadrant of the TM at 135.7 µm, and the lowest in the anterior region at 109.2 µm [28].

## 2. Tympanic Membrane Perforations and the Problems They Pose

TM perforations affect millions of people worldwide, and can have multiple causes, such as accidents, assault, hot and sharp objects and during cleaning with such, acoustic traumata, or middle ear infections [29,30,31,32]. TM perforations are differentiated into acute and chronic perforations. While most acute TM perforations heal without interference and in about 80% of cases spontaneously, chronic TM perforations are termed such if they fail to heal within three months [16]. According to TM surface loss during perforation, TM perforations can be additionally classified into small (≤25%), medium (≤50%), subtotal (≤75%), and total (≥76%) perforations. Another classifier for TM perforation is location; they can be classified as anterior, posterior, median, marginal and attic perforations. Sound conduction disturbance is especially significant with perforations at the umbo and manubrium site of the TM and additionally increases with an increasing size of the perforation, while becoming increasingly difficult to self-heal [33,34].

TM perforations may lead to significant morbidities, such as hearing loss [35], cholesteatoma [36], chronic ear discharge [16], or recurrent infections, where chronic otitis media represents one of the most common infectious diseases, especially in young children [37,38]. Regeneration failures of the TM leading to chronicity may arise from several factors, including epithelisation of the perforation margins, barring further healing, infections such as otitis media or inadequate growth factor supply at the TM perforation rim and thus a dysregulation of inflammation, re-epithelialisation, angiogenesis and subsequent tissue regeneration [39]. During TM wound healing, the tissue first undergoes the first two sequential stages of conventional wound healing; haemostasis and inflammation. Then, opposed to other tissues of the body, cell migration proceeds cell proliferation and occurs mid-air; the squamous epithelial layer forms bridges across the wound, followed by the remainder or the epithelial layer and concluded by the re-formation of the fibrous layer [16,40,41,42]. However occasionally, the fibrous layer fails to migrate, leading to a dimeric TM replacement tissue, only composed of random collagen fibres, instead of the radial and circular fibre arrangement [43]. The lacking of a fibrous layer results in a more flaccid TM with poor sound conduction [16]. Additionally, since the wound healing of the TM must occur suspended in air, the formation of the new tissue lacks structural support from any underlying tissue or matrix [44].

To close chronic TM perforations and restore TM integrity and function, there currently are two therapeutic methods: myringoplasty and tympanoplasty [45,46]. Tympanoplasty is a surgical procedure which includes TM manipulation and thus reconstruction, and, if necessary, eradication of disease from the middle ear; if it is done with inspection of the ossicular chain, it is known as tympano-ossiculoplasty. Myringoplasty is limited to the drumhead and neither includes manipulation of the ossicular chain, nor the middle ear [47]. The most commonly used grafts are made from autologous materials; the materials are harvested from including temporalis fascia representing the gold standard, perichondrium, fat and cartilage [23,48]. The graft, however, mainly serves as a guidance and scaffold for keratinocytes across the wound [23]. For graft attachment, the anterior and marginal, as well as sub-total and total TM perforations offer less area for the surgical materials to hold and are thus more challenging to close [33]. As autologous grafts are always accompanied by surgery at the donor site, they come with corresponding risks and are not available in unlimited quantities, should the TM perforation return [23]. Limitations of autologous grafts present as possible occurring donor site morbidity and a lack of transparency for future check-ups. Additionally, quality in biomechanical and acousto-mechanical material properties is lacking when compared with the native TM [49,50,51]. Attempting to develop a more cost-effective method, the paper patch method was introduced in 1887, where the patch guides the migrating epithelium as a scaffold [52,53]. It was, however, found that the paper patch method is not suitable for perforations larger than 3 mm [54] and only about 50% of chronic perforations may close with a paper patch being used [55]. Further, paper patches were found to be non-biocompatible, non-transparent, non-flexible, easily detaching and not resistant to infection [55].

To offer an alternative to the autologous graft materials and paper patches and to circumvent surgery, different materials, such as cadaveric, xenogeneic, or nonorganic sources have been proposed [16,20,23].

## 3. Scaffold Requirements for Tympanic Membrane Replacements

Scaffolds are described as three-dimensional structures with a porous, fibrous and permeable architecture made from corresponding biomaterials. As the scaffold should eventually be completely degraded and replaced by neo-tissue, the material should be biodegradable in an acceptable time frame and neither the polymer nor its degradation by-products should elicit any inflammation or toxicity in vivo. Thus, they may serve as temporary platform for tissue defect reconstruction. A porous surface should better permit cell adhesion and promote cell growth, while at the same time allow cultivation of cell differentiation. Pore size should be large enough to provide space for cell adhesion and proliferation, as well as the formation of new ECM, while at the same time offer minimal diffusion constraints for cellular needs [56,57,58]. Additionally, they may transport bioactive materials such as drugs, inhibitors and may serve as mechanical barrier against infiltration from new forming native tissues in order to prevent tissue regeneration and restoration [59]. A graft however, is any tissue or organ used for transplantation or implantation and needs thus be demarcated from a scaffold [16].

### 3.1. Requirements for Clinical Application

As a synthetic scaffold stands in stark contrast to an autologous graft, first and foremost a tympanic membrane scaffold should be able to be obtained in a quick and easy fashion, and, opposed to the limited availability of autologous grafts, be available in unlimited quantities [60]. If available of shelf, a TM scaffold is advantageous in revision cases, significantly reducing surgery time and cost. It would be advantageous to manufacture a scaffold in various thicknesses to specifically tailor the scaffold to patient needs [16]. During surgery, handling properties of a TM scaffold define usability; good cutability enables matching of the scaffold to meet patient-dependent criteria, without losing any functionality of the scaffold [61]. For further examinations post-surgery, a transparent TM scaffold is desirable, allowing direct visualisation of the middle ear to identify any possible complications and to enable monitoring of the repair progress [16].

### 3.2. Biological Scaffold Requirements

For TM scaffolds, additional criteria to general scaffold requirements need to be met, as the TM hangs suspended in air and specific material parameters are needed to ensure a good hearing quality after implantation. The native TM exhibits a conical shape. It has been suggested that this very shape plays an important role in transmitting sound to the cochlea [4,62]. Biomechanical and sound conduction features of the TM are largely based on the specific, anisotropic arrangement of collagen fibres; for TM grafts it would be preferential and beneficial, to imitate this arrangement to further improve the acousto-mechanical properties [33,63]. It is known that keratinocyte migration dynamics are highly regulated by the host environment; furthermore, biochemical signalling including communication with other dermal cells is crucial to cell migration and proliferation. Specifically, collagen type I plays an important role in keratinocyte migration [64]. As the TM exhibits a highly organised network of radial and circumferential collagen fibres, at least some of the keratinocyte migratory pattern may be approximated by these [65]. In hindsight, it becomes crucial for a TM graft to closely mimic these arrangements of collagen fibres. Cellular proliferation zones are located in the central and superior regions of the PT; keratinocytes migrate from there inferiorly and radially outward [64]. A beneficial migratory microenvironment for keratinocytes would thus aid the healing of TM perforations [65].

### 3.3. Characterisation of Tympanic Membrane Scaffolds

For a material to be considered as potential scaffold for tympanic membrane replacement, several criteria and tests need to be met. In the following section, these are presented.

FE analysis. For extending knowledge of middle ear mechanics, finite element (FE) modelling is an important part in a scientist’s toolkit [66]. FE describes a method where a system is divided into smaller, more simple elements; their analysis via computer simulation finds application in biomechanical analysis and engineering, providing a useful tool to accurately simulate anatomical structures such as the middle ear and the delicate structure of the tympanic membrane [67]. Such a model aids in better understanding middle ear physiology in normal and diseased conditions, and for designing TM scaffolds and grafts with considerations to ideal specifications [68]. Models predicting biomechanical behaviour need appropriate material parameters to accurately represent tissue behaviour. However, FE simulations find it difficult to accurately address the inherent complex anisotropic, nonlinear and viscoelastic characteristics of native tissues, especially under dynamic loads, possibly resulting in mistakes [69]. Ideally, each human body FE model would use personalized data, but there are numerous barriers to doing so. In general, it has been easier to obtain data from animals than from humans, as testing with animals is easier for material property collection resulting in a dearth of material properties representing human tissue [70]. Additionally, simulations for soft tissue models based on complex nonlinear multi-physics equations come with the expense of high computational costs and running times [71]. As such, Fay and collaborators have firstly combined the anatomical collagen fibre arrangement in the TM describing it in a mathematical model of the middle ear and ear canal, showing that an ideal fibre arrangement induces better vibration in a frequency range of 200 Hz to 20 kHz [72].

Tensile measurements. As a scaffold is intended to be used temporarily until its replacement by neo-tissue, it is important that the scaffold furthermore withstands the loads and stresses of the native tissue it temporarily replaces. It therefore is imperative to evaluate rheological parameters, including tensile strength (the maximal stress a material withstands before breaking) and the elastic modulus (the measured strain in response to an applied tensile or compressive stress along the force) [73,74]. Tensile strength gives rise to Young’s modulus, which describes the ability of an elastic material to resist deformation to an applied stress [75]. The PT region is the most frequently damaged, with a TM thickness of 135.7 µm in the posterior superior quadrant and 109.2 µm in the anterior region. [28], so an ideal TM scaffold should range between these two values in thickness. For good hearing post-surgery, the TM scaffold should have the same Young’s modulus as a native TM between 20 to 60 MPa [24,25].

Water Contact Angle measurements. Surface properties, such as topography and wettability, are crucial for cell adhesion, differentiation and proliferation [76]. The wettability of a scaffold can be altered using various chemicals and topological modifications. It has been accepted that a higher water contact angle stands for a material with higher hydrophobic properties, while a smaller one stands for a more hydrophilic material [77]. In general, mammalian cells interact better with hydrophilic surfaces [78], but typically hydrophilic surfaces lower protein affinity [79]. Further, protein conformation is strongly affected by interfacial adsorption and the corresponding material and thus directly influence interactions between cells and substrate [80]. When biomaterials interact with their environment after implantation, they are typically reached by proteins before cells start to populate the scaffold surface; cells migrate on the scaffold surface via mediation of the beforehand adsorbed protein layer [81]. To measure this, the water contact angle is used [79] and a surface is considered hydrophobic when measurement results are between 150 and 90°, hydrophilic if between 90 and 10° [80].

Porosity. An important factor for cell seeding behaviour is the porosity of the given scaffold, as a porous structure is necessary for mass-transport of nutrients for cell nutrition, cell migration and cell attachment [82]. The most common characteristics of porous materials are the specific surface area, the average pore-size and its distribution [83]. To determine these characteristics, the experimental methods include following: During Archimedean porosimetry the scaffold is dried, then immersed in water or ethanol and subsequently the porosity is calculated via the dry, wet and immersed weight [84]. Another method is the mercury intrusion porosimetry together with the Washburn equation [85]. Characterisation of porosity is especially important, as it has been reported that keratinocyte attachment to scaffolds is dependent on pore size, with best attachment to 3 µm pores [86], thus benefitting cell seeding on a TM scaffold.

Cell culture tests. As a next step to test a TM scaffold, cell culture tests are important to test biocompatibility of the investigated scaffold, as well as cell migration, adhesion and proliferation. Special attention shall be paid to keratinocytes. TM proliferation, in contrast to other epidermal sites, is not uniform, but restricted to a few sites on the edges of the TM. Due to its in air suspended nature, keratinocytes do not proliferate from ground up, but rather migrate from the rim of the TM inward [87]. Frumm and collaborators suggested that keratinocytes thereby follow clues from the collagen structure of the TM, which is lacking in chronic TM perforations [65] and should, ideally, be supplied by the scaffold. Alternatively, human mesenchymal stem cells (hMSCs) have been reported to be used as model primary cells for keratinocytes [88]. To verify the ability of scaffolds to support such notions, cell migratory tests, adhesion tests and proliferation tests need to be performed.

Animal Studies. As novel materials have an unknown safety and efficacy profile in living organisms, animal studies become necessary for evaluation of potential risks, before a clinical trial can start [39]. For testing TM perforation with newly designed scaffolds, several established animal models exist; the most prominent model is the chinchilla, as it has a similar TM diameter and a similar hearing frequency to the human TM and a large hearing canal permitting good access to the TM. Chinchillas however, are expensive to house and often come from strained environments into research [89]. In contrast to the chinchilla model, rats are more inexpensive, and easier to house. However, their TM is semi-occluded and smaller in diameter than in humans, and they frequently get infected with otitis media [39,90]. The ears of guinea pigs have a similar anatomy to the human ear, with a more robust temporal bone than in rats and can be more easily handled in surgical experiments concerning stapes, TM and oval window [39,90]. Further, to properly evaluate healing of a chronic TM perforation with the new scaffold, a corresponding perforation with chronic character must be created. For this, various methods such as the infolding technique or delayed healing by chemicals have been proposed. However, for a more detailed review on animal studies in chronic TM perforation studies, the attentive reader shall be referred to the detailed work by Wang and collaborators [39] at this point.

Cadaveric Temporal Bone testing. To verify the possibility of implantation in a realistic setting, and the ability of the scaffold to close TM perforations and to further restore hearing, TM reconstruction and surgery can be simulated in a human cadaveric temporal bone. In such a setting, the surgical conditions, handling, duration and necessary tools during surgery can be evaluated in a close to reality simulation. In a further step, vibration velocity can be measured in a human like setting [61], thus providing a realistic application of the scaffold, which is a limitation of animal studies. A limitation of human cadaveric temporal bone testing, is however long-term studies, as cellular migration, scaffold degradation and long-term adaption are not possible due to the cadaveric state of the temporal bone.

Laser Doppler Vibrometry. Laser Doppler Vibrometry (LDV) uses the Doppler shift principle to determine the immediate velocity of a moving object. For this, the frequency of the emitted light by a laser is compared to the frequency of the laser’s light reflected by the moving object, enabling non-contact measurements of vibration velocity. As such, LDV is the method of choice to investigate the motions of the TM and has been used to measure middle ear velocity in animals, human cadaveric temporal bones, and living humans [91,92]. LDV therefore presents a good choice for determine TM scaffold properties in applied cases.

## 4. Manufacturing Techniques for Tympanic Membrane Scaffolds

The fibrous ultrastructure of the TM consists of highly ordered collagen fibres, which are largely responsible for mechanical hardness [23]. Especially, the radial collagen fibres have been suggested to play an integral role in effective high frequency sound conduction. As conventional autologous grafts do not restore the microanatomy of a native TM, hearing after surgery may be lacking [93]. Previous results have indicated that geometrical features of scaffolds exhibit a significant influence on cellular distribution, alignment and later collagen deposition, leading to a TM scaffold with comparable acoustical attributes to a native TM [63]. Considering this, the need for a close mimicking and remodelling of the ultrastructure of the native TM and its ECM becomes apparent. To solve this, two different approaches may be used; scaffolds produced by additive manufacturing or textile-based technologies [94]. In the following, both approaches are presented shortly and their according benefits are highlighted. Table 1 lists the different scaffold requirements and the corresponding values taken from the later reviewed literature on TM scaffolds.

### 4.1. Additive Manufacturing for Tympanic Membrane Scaffolds

Additive manufacturing, also called 3D-printing, is an approach, where 3D designs are built directly from computer-aided design (CAD) models, without further need for part-specific tools. During manufacturing, multiple layers are built in the X-Y direction by adding material in a layer-wise fashion on top of each other to generate the Z dimension. The model thereby follows a predefined geometry based on the beforehand built CAD model. As soon as the designed part has been built, it can be used for functional prototypes or in practice [95,96]. The commonly used printing methods are fused deposition modelling (FDM), selective laser sintering (SLS), light curing technology (SLA), the ink-jet printing technique and two-photon polymerisation (TPP) [97]. Implementation of additive manufacturing in medical applications enables the fabrication of medical devices used in diagnostics and surgeries, as well as in ortheses and protheses. These approaches benefit immensely from designing and printing personalised structures, tailored to the individual patients need [98]. Additive manufacturing makes it possible to build complex structures and geometries with high precision, reproducibly and accurately [99]. In context of TM scaffolds, additive manufacturing enables the precise manufacturing of the collagen structure of the native membrane [100].

### 4.2. Fibre-Based Scaffolds as Tympanic Membrane Scaffolds

Fibre-based structures in general offer many favourable properties for biological applications. Their high specific surface area and high porosity enables a more effective tissue interface, better drug delivery and cell attachment, interaction with the surrounding material and close mimicking of the ECM. Being lightweight and easy to fold allows for a minimal invasive implantation, while attributes such as strong and flexible may better tolerate surgery-induced damage [101,102]. Their fibrous structure especially bestows mechanical stability and elasticity to the fabricated scaffold. Textile fabrication techniques allow a high degree of precise control of porosity and fibre orientation, contributing even further to biomimicking the original tissue [103]. As such, the most common techniques are spinning methods such as wet and dry spinning, melt spinning, gel spinning and electrospinning [102], but knitting has been applied as well [104]. With further advances in regenerative medicine, fibre-based scaffolds and the different fabrication techniques offer various possibilities to engineer different types of scaffolds for biological tissues, proposing alternatives to harvested tissues and autologous implants [104], having been used in applications such as hernia mesh repairs, heart valves, vascular grafts, drug delivery systems, wound dressings and more [105]. Especially in context of the TM, whose acousto-mechanical properties largely depend on the fine tuning of the ECM and the collagen ultrastructure, textile-based scaffolds and textile manufacturing techniques with their high tunability of structure, porosity and fibre orientation become the apparent choice for a scaffold. This is true especially for nonwoven materials; nonwovens are classified as ‘engineered fibrous assembly, primarily planar, which has been given a designed level of structural integrity by physical and/or chemical means, excluding weaving, knitting or papermaking’ [106]. Due to their ease of processing and huge versatility, nonwovens are advantageous materials for scaffold production and can furthermore be adapted into providing different structures and fulfilling different functions [107] and thus shall be the focus of this work.

#### Electrospinning

One of the most common methods to produce nonwovens is electrospinning [107]. During electrospinning, nanoscale fibres are created by jetting and subsequent mechanically stretching liquefied polymers by electrostatic forces [108]. This results in non-woven membranes with fibre thicknesses ranging down to just a few nanometres, larger surface areas and higher porosity than fabrics obtained by traditional spinning techniques [109]. The major components of a typical electrospinning setup are a high power voltage supply, a spinneret connected to an electrospinning fluid supply system and a grounded collector plate [110]. The polymer solution is transported to the spinneret via the supply system, typically consisting of a syringe and a microinjection pump. At the nozzle, the building droplets are deformed by the interaction of electric field force and surface tension. As soon as the electric force is equal to the surface tension of the polymer solution, the charged hemispherical droplet hangs at the tip of the nozzle in a balanced state. As the voltage and thus the electrical field is increased, the droplet is gradually stretched into the Taylor cone. Once the threshold voltage is reached overcoming surface tension, droplets are ripped from the tip of the Taylor cone by the electrical field and the solution is jetted towards the collector. The initial jet shoots forward in a straight line and then experiences a whipping motion due to bending instabilities, while the solvent starts to volatilise. Thus the jet elongates into thinner diameters by stretching and motion until it is finally deposited on the collector in form of nanofibers [108,111].

The resulting membrane has a remarkable morphological resemblance to the ECM and especially to collagen fibres, with a high surface area to volume ratio presented by highly interconnected pores, ensuring an environment favourable for cell attachment and oxygen and nutrient transport [112,113]. So far, electrospun membranes have been used in various biomedical applications, including wound dressings [114], bone tissue engineering [115], engineered skin [116] or vascular grafts [117]. Further, electrospinning has also been proposed for TM scaffold fabrication, due to its high likeliness to the ECM and its other favourable properties [49]. It has to be noted, however, that solvents used in electrospinning are often toxic and may leave toxicity and chemical residues in the spun fibres behind, if not treated properly [118].

### 4.3. Melt Electrowriting

At the intersection of textile-based technologies and additive manufacturing lies melt electrowriting [119]. This technique processes microscale polymer fibres into high-resolution and well-organised scaffolds, resembling the architecture of native tissue. Basic components of a melt electrowriting setup comprise a heated polymer feeding system, a three-axis positioning configuration enabling depositioning in every axis, and a high voltage source. Similar to FDM, a polymer is molten and then extruded through a nozzle at a temperature above its melting point. An electrical field is applied, leading, similar as during electrospinning to the formation of a Taylor cone. The experienced temperature decreases once the melt leaves the nozzle and the Taylor cone facilitates the rapid solidification of the material, resulting in well-rounded fibres, while the applied charges minimising electrodynamic instabilities. The jet shoots vertically towards the collector, enabling the precise control characteristic for additive manufacturing in the positioning of the jet by adjusting the relative position of the printhead. Printing speed can be further adjusted to modify the morphology and shape of the resulting structure, resulting in fibres with a minimum feature size of 800 nm. It is then written onto a collector in a layer-wise fashion [120,121,122,123].

**Table 1:** Materials prepared by the presented different manufacturing methods and their corresponding scaffold properties. FDM: fused deposition modelling; ES: electrospinning; MEW: melt electrowriting; BC: bacterial cellulose; SFM: silk fibroin membrane; YM: Young’s modulus; TSt: tensile strength; TS: tensile stress; WCA: water contact angle; TM: tympanic membrane; n.a.: not available.

Method	Material	Mechanical Properties [MPa]	Thickness [µm]	WCA [°]	Experimental Acoustic Properties	Porosity	Ref.
FDM	PLA	TSt: 4.72	80–616	n.a.	Similar motion patterns at >1000 Hz, different at 400 Hz	n.a.	[100,124]
	PCL	TSt: 9.4–10.2	592–616	86.5	Similar motion patterns at 1000 Hz, different at 400 Hz, similar to fascia	n.a.	[100,125,126]
ES	PLA	YM: 0.08–203.7 TS:2.5–5.4 TSt: 1.0–1.6	13.5–90	78–112	n.a.	86–91%, pore size 3–30 µm	[88,127,128]
	PEOT/PBT	YM: 68–80	100	n.a.	n.a.	n.a.	[129,130,131]
	PVA	YM: 99.27–729.11 TSt: 6.79–27.06	18	47–88	n.a.	Pore size: 224.8–1380 nm	[132,133]
	PCL	YM:0.7–35.87	7.31–74.2	71.8–74.2	Similar, fibre mimicking enhanced acoustic response	n.a.	[61,127,134]
ES/FDM	PEOT/PBT	YM: 6–13	80–384	n.a.	Similar to native TM	79.2–80.8 Pore size: 0.003–300 µm	[63,88]
MEW	PCL	n.a.	40–450	n.a.	Similar acoustic behaviour, close resonance peaks to native TM	Fibre spacing: 100–500 µm	[123,135]
Membranes	BC	YM: 11.51–12.47 TS: 11.37–12.33	9.75–10.91	45.6–52.2	n.a.	n.a.	[49,136]
	SFM	TS: 9.7	33	31.17–36.45	n.a.	n.a.	[137]

## 5. Different Materials for Electrospinning TM Scaffolds

### 5.1. Silk Fibroin-Based Scaffolds

Silkworm silk, as produced by domesticated *Bombyx mori*, has been used in Chinese textiles for more than 4000 years [138], based on its lustre, lightweight, flexibility and mechanical strength [139,140]. In the biomedical context, silk has been used for sutures for centuries [141], and has, in a more recent time period of silk usage, been approved by the FDA for selected biomedical devices [142]. Raw silk consists of a fibrous core protein, fibroin, surrounded by sericin, a gumming protein [143]. For further biomedical use, raw silk has to be degummed to obtain the pure silk fibroin fibres, as contamination of fibroin with residual sericin is reported to be the main cause for eliciting immune response activation in virgin silk [144,145]. Conversely, sericin on its own appears to be harmless and inert in solution in biomedical contexts [146]. Silk fibroin as a whole can be subdivided into light (≈26 kDa) and heavy chains (≈391 kDa), linked C-terminally by a single disulphide bond [147]. The foundation of the unique stability and strength of fibroin lies in the composition of the heavy chain, which is composed of twelve repetitive hydrophobic domains, each separated by eleven shorter, hydrophilic linking sequences. These hydrophobic domains form the main structural components of fibroin as crystalline and semi-crystalline β-sheets [148,149,150].

As scaffold material, silk fibroin offers a range of benefits when compared to other scaffold materials. As important for all scaffolds, silk fibroin scaffolds are degraded in vivo, with degradation to completion ranging from two and six months (all-aqueous prepared scaffolds) to one year (organic solvent prepared scaffolds) due to macrophage degradation [151]. Degradation products of silk fibroin present as amino acids and peptides and are easily resorbed in vivo [152]. Due to its robust β-sheet structure, the need for further, possible toxic crosslinking can be avoided [151], as for example is often the case with collagen scaffolds [94]. Silk fibroin membranes can be obtained by degumming native silk from *Bombyx mori* cocoons, dialysing the obtained silk fibroin and lyophilising it for drying. The dry silk fibroin is then dissolved in formic acid or trifluoroacetic acid and dried to obtained membranes [49].

Table 2 lists scaffold material, mechanical properties, thickness, water contact angle, acoustic properties and porosity of the described silk fibroin membranes. In Table 3, in vitro tests in regards to TM, silk fibroin membranes are compared with adhesion and proliferation, migration, orientation and collagen analysis of the chosen cell type.

The commonly used paper patch was compared to a silk fibroin membrane (SFM) by Kim and collaborators in Sprague-Dawley rats, detailed in Table 4. Investigated silk patches were found to be hydrophilic. The group found that the SFM better adheres to the perforated edge of the TM than the paper patch, resulting in a shorter healing time after perforation closure 7.2 ± 1.48 and 9.1 ± 1.11 days respectively and a better organisation of the healed area [136]. Complementary, Levin and collaborators investigated the growth of human tympanic membrane keratinocytes on SFM scaffolds using immunostaining. The group found that the scaffolds supported growth, adhesion (expression of occluding and zona occludens 1 protein) and proliferation (expression of proliferation marker MIB- 1) of these cells, while maintaining their original cell lineage (expressing Epithelium-specific Ets-1), further cementing the use of silk fibroin in tympanic membrane applications [49]. Biodegradation behaviour of the SFMs used for TM perforations was evaluated by the group of Lee by subcutaneous implantation in Sprague-Dawley rats. Thickness of the implanted silk membranes decreased gradually, with the thickness being 65% of the original after 19 months. SEM analysis showed that SFMs began decomposing and started to crack six months post-implantation, and after a year, fibroblast and collagen fibres were observed. Nano-particles of the cracked silk fibroin were absorbed into the surrounding tissue, barring any inflammatory response [153].

Lee and collaborators compared SFM to the paper patch method in 52 patients with traumatic TM perforations in a clinical study. The SFM showed generally better mechanical properties and reduced healing time; closure time in the SFM group was found to be 13.7 ± 4.7 days, with the paper patch group being slightly slower with 16.7 ± 4.1 days [154]. Based on these results, the same group also performed a cohort study comparing SFMs with conventional perichondrium myringoplasty in 40 patients. SFM application resulted in lower otorrhea, minor complication rates and high patient satisfaction. However, hearing results were not significantly different between the two groups, but the hearing gain was higher in the SFM group. Total surgery time for the SFM group was significantly shorter with 13.7 ± 4.96 h compared to 29.5 ± 7.36. The SFM was easier to manipulate [155]. The transparency of the SFM in both cohort studies was found to ease post-surgery controlling and was advantageous in deciding when to remove the patch [154,155]. Clinical studies are summarised in Table 5, in terms of perforation type, scaffold material used, closure time, hearing gain and surgery time.

### 5.2. Bacterial Cellulose-Based Scaffolds

**Figure 3:**
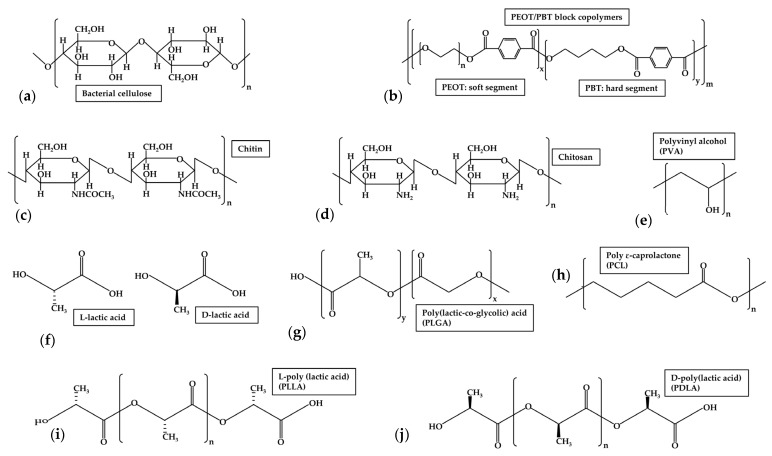
Structural formulas of the presented materials. Detailed are (**a**) bacterial cellulose, (**b**) PEOT/PBT: block copolymer, (**c**) chitin, (**d**) chitosan, (**e**) polyvinyl alcohol (PVA), (**f**) L-lactic acid and D-lactic acid, (**g**) poly(lactic-*co*-glycolic) acid (PLGA), (**h**) poly ε-caprolactone (PCL), (**i**) L-poly(lactic acid) (PLLA) and (**j**) D-poly(lactic acid) (PDLA).

Bacterial cellulose (BC) is a natural polymer produced by different genera of bacteria, including *Komagateibacter*, *Escherichia*, *Sarcina*, *Pseudomonas*, *Agrobacterium*, and *Rhizobium* [160,161]. BC is structured in two distinct regions: a crystalline region consisting of highly ordered, parallel cellulose strands, and an amorphous region comprised of randomly organised fibres (Figure 3a) [162]. Its chemical structure is similar to plant cellulose, composed of β-1,4-glucan chains, which are assembled into a dense and interconnected network composed of ribbon-like structures. Due to this, BC exhibits a high aspect ratio with a high surface area and high porosity [163,164]. Extensive studies regarding BC biocompatibility have demonstrated its low cytotoxicity and excellent biocompatibility, and that it does not induce inflammatory responses or toxicity when in contact with living tissue [165,166]. In the biomedical field, BC finds application in fields including wound dressings, tissue engineering scaffolds or artificial blood vessels [162,167]. Especially for TM scaffolds and regeneration, the transparency of BC is beneficial, as well as its ability to enhance tissue regeneration. Thus, BC may successfully mimic the structure of the ECM, and guide the proliferation of tympanic cells to the site of perforation, supporting cell growth of all three layers of a native eardrum, while its dense nanofibrillar network secures the middle ear from infections. The for the TM important Young’s modulus of BC is dependent on its fibre length [168].

Table 1 details mechanical properties, thickness, water contact angle, acoustic properties and porosity of BC-based scaffolds. In Table 2, in vitro tests in regards to TM scaffolding are compared with adhesion and proliferation, migration, orientation and collagen analysis of the chosen cell type.

Kim and collaborators fabricated a BC membrane from *Gluconobacter xylenes.* In vitro studies showed that cell proliferation of keratinocytes and fibroblasts were significantly stimulated by the presence of the BC membrane. In vivo studies in Sprague-Dawley rat with traumatic TM perforations successfully validated that BC membranes accelerated healing time, also resulting in a thicker TM with denser, although more irregular collagen fibre arrangements (Table 3). When compared to unassisted healing, the regenerated TM was thinner. Both cases were confirmed to exhibit three tissue layers; possibly giving rise to a better sound conduction in the BC membrane group, due to its denser nature [137]. Biskin and collaborators used BC membranes for myringoplasty in 12 patients with a total of 16 ears, with follow-up controls of the post-operative condition in the range of 6 to 24 months. At six months, the perforation was completely closed in 13 ears, while persisting in three, thus proving to be effective and safe in small perforations [156]. The randomised controlled trial of Silveira and collaborators was conducted with 40 patients with TM perforations secondary to chronic otitis media and treated with temporal fascia or BC membranes. Healing time was similar in both groups, but surgery time was lower in the BC membrane treated group with 14.06 ± 5.23 min compared to 76.50 ± 17.92, leading to a great cost reduction of 93% (Brazil) [157]. Mandour and collaborators compared the efficacy of a BC membrane with a conventional fat graft in TM perforation closure as control. 120 patients underwent myringoplasty with small to moderate sized perforations. The study showed healing in all patients with small perforations in 3.11 ± 0.84 weeks and 85% healing in patients with moderate sized perforations in 5.03 ± 0.69 weeks (compared to healing time in the group treated with fat grafts of respectively 4.97 ± 0.68 and 7.07 ± 0.95 weeks) with a mean surgery time of 15.01 ± 0.46 min and a post-surgery air-bone gap of 6.68 ± 0.29 dB. The study demonstrates the great efficacy of BC membranes for TM perforation surgery and mending, as healing occurred in shorter time compared to the conventional fat graft [158]. The study of Pinho and collaborators used a BC film in 24 patients with traumatic perforations of the TM and evaluated results using otoscopy and audiometric examinations. After BC film application, symptoms of tinnitus, ear fullness and autophony were relieved and immediate and significant improvement in tonal thresholds was observed for all frequencies except 8000 Hz, highlighting a recovery of TM function [159]. Clinical studies are summarised in Table 4, in terms of perforation type, scaffold material used, closure time, hearing gain and surgery time.

### 5.3. PLA-Based Scaffolds

Poly(lactic acid) (PLA) is a biodegradable polyester (bioplastic) produced from lactic acid from renewable resources, such as food wastes as primary raw materials source [169,170]. It is a linear aliphatic polyester, with similar physical and mechanical properties to polyethylene terephthalate (PET) [171]. After polymerisation, two kinds of PLA may assemble: PDLA and PLLA (Figure 3f,i,j) [172]. PLA and its copolymers are generally biocompatible and degrade after implantation; toxicity due to accumulation of acidic degradation products, such as lactic acid, is a possible concern, due to its pH of 4 to 5. They are rapidly neutralised by local body fluids and as such usually do not affect biocompatibility [173]. PLA is characterised with a high tensile strength (Young’s modulus of 3.84 GPa [171]), stiffness, hardness and thermoplasticity [124]. Table 6 gives a summary and comparison of the scaffolds prepared from PLA, based on mechanical properties, scaffold thickness, water contact angle, acoustic properties and porosity. In Table 7, in vitro tests in regards to TM scaffolding based on PLA are compared with adhesion and proliferation, migration, orientation and collagen analysis of the chosen cell type.

#### 5.3.1. Additive Manufacturing of PLA-Based Scaffolds

Kozin and collaborators designed and fabricated PLA-based scaffolds, infilled with a fibrin-collagen hydrogel mimicking the extracellular matrix. The produced scaffolds exhibited a reproducible acoustic behaviour, resisting deformation better than temporalis fascia and showing a similar behaviour to the native TM [100]. Based on this study, Rostam-Alilou and collaborators carried out a vibro-acoustic analysis of the proposed PLA-scaffolds in silico (Table 7), showing that they are in significant correlation at 1000 Hz frequency to the human TM, while exhibiting the most significant displacement magnitude at all frequencies, when compared to PCL-based scaffolds [125]. FE modelling of different scaffold materials is summarised in Table 8. Ilhan and collaborators fabricated 3D-printed PLA-based scaffolds in combination with chitosan in various concentrations to obtain more biocompatible scaffolds, however resulting in a decrease of tensile properties. The produced scaffolds showed similar thickness and mechanical properties to the native TM [124].

#### 5.3.2. Electrospinning of PLA-Based Scaffolds

**Figure 4:**
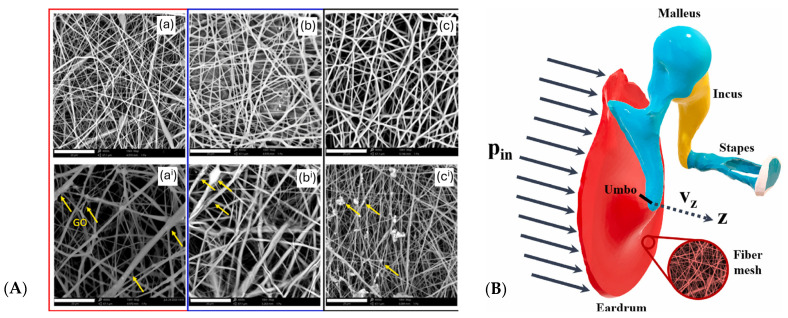
(**A**) Morphological analysis of electrospun membranes. Top row shows neat polymers PCL (red), PLA (blue) and PLGA (black). Bottom row shows nanocomposites made of the polymer loaded with graphene oxide: PCL/GO (red), PLA/GO (blue) and PLGA/GO (black). Scale bar indicates 20 µm. (**B**) in silico model of the middle ear for characterising the nanocomposites constituting the tympanic membrane. (Adapted from [127], under a CC-BY 4.0 licence).

Milazzo and collaborators used PLA and PLLA for electrospinning. The addition of graphene oxide resulted in stiffer membranes with Young’s moduli of 53.0 ± 11.3 MPa and 189.0 ± 14.7 MPa respectively. The acoustic behaviour of the membranes was investigated in silico using FEM analysis of the middle ear, showing that the effect of added graphene oxide was rather small for the eventual stapes velocity response. PLA based materials rendered the best results, therefore making it the most suitable TM graft material (Figure 4A,B) [127] (Table 8). Mota and collaborators used poly(lactic-*co*-glycolic) acid (PLGA) for the fabrication of electrospun membranes on a TM patterned collector, resulting in a mesh thickness of 24.61 ± 8.15 µm, with a measured height for the pattern of 26.22 ± 12.71 µm. Tests with mesenchymal stem cells showed low viability, but penetrated the fibre mesh. As the membrane was found to be very delicate at handling and thin, it barely satisfied minimal requirements for mechanic stability of the TM and negatively affected cell viability [88]. Immich and collaborators also studied PLGA/PLLA (50:50 blend ratio) electrospun meshes. Ultimate tensile strength was found to be at 1.3 ± 0.3 N and Young’s modulus at 0.08 MPa, making the scaffold soft and easily deformable for the patient’s need. Fibroblasts and keratinocytes both showed proliferation on the scaffold and were able to proliferate in a coordinated manner when seeded in co-culture. The authors performed in vivo measurements using Sprague-Dawley rats (Table 3) and a more natural membrane with a high possibility of natural function was observed when the electrospun membrane was used. Tests to verify this, however, where not performed [128].

### 5.4. PEOT/PBT-Based Scaffolds

Polyether-ester multiblock copolymers poly(ethylene oxide terephthalate)-poly(butylene terephthalate) (PEOT/PBT) belong to the family of thermoplastic elastomers, with PEOT being classified as the soft segment and PBT as the hard segment (Figure 3b) [175]. This especially is relevant for the strength of the material, as the hard PBT segments exhibit a glass transition temperature above body temperature and thus are able to crystalise. The below room temperature glass transition temperature of the hydrophilic, soft PEOT provides the flexible and hydrophilic attributes to the copolymer system [176]. As its degradation rate can be tuned depending on the application and its wettability is higher compared to PCL or other biodegradable thermoplastics such as PLA, it has become a competitor for scaffold engineering applications [177]. Table 9 gives a summary and comparison of the scaffolds prepared from PEOT/PBT, based on mechanical properties, scaffold thickness, water contact angle, acoustic properties and porosity. In Table 10, in vitro tests in regards to TM scaffolding based on PEOT/PBT are compared with adhesion and proliferation, migration, orientation and collagen analysis of the chosen cell type. Danti and collaborators fabricated PEOT/PBT electrospun membranes. They exhibited a thickness of 220 ± 56 µm and a porosity of 80 ± 0.8%. Human TM keratinocytes were found to be viable and to adhere to the scaffold after 48 h of culture time [129]. Mota and collaborators used PEOT/PBT additive manufacturing techniques, mimicking the collagen structure and fabricating a biomimetic scaffold in one or two layers with an additional electrospun layer in either case. hMSCs adhered well to both fabricated scaffolds and penetrated well into the electrospun mesh, as shown by histological analysis. However, the authors found that only the triple layered scaffold permitted biomimetic cellular disposal and retracing of the collagen arrangement of the native TM, highlighting the need for a structured TM scaffold [88]. Based on this two studies, Anand and collaborators investigated the scaffold geometry in silico of PEOT/PBT scaffolds deconstructed into radial and circumferential fibre designs using FDM and electrospinning (Table 8). The results obtained by FEM suggest a geometrical dependency on the mechanical and acoustical response, with the effect of the radially aligned fibres being more prominent. Subsequent studies of the simulated and beforehand described electrospun with hMSCs and neonatal human dermal fibroblasts (NHDFs) also revealed a positive influence of the simulated hierarchy on cellular alignment and subsequent collagen deposition. Based on LDV measurements, the fabricated scaffolds were comparable to the native TM, further highlighting the influence of scaffold geometry for a close to native function [63].

#### PEOT/PBT/Chitin Nanofibrils Based Scaffolds

Poly (β-(1→4)-*N*-acetyl-D-glucosamine), more commonly known as chitin, is a natural polysaccharide, synthesised by a great number of invertebrates such as sponges, molluscs, nematodes, arthropods, but also fungi or yeasts, making it the second most abundant biopolymer after cellulose [178,179]. For biomedical applications, chitin presents strong antibacterial effects, biocompatibility, non-toxicity and a high humidity absorption [180].

Danti and collaborators fabricated PEOT/PBT copolymer in combination with chitin and poly(ethylene glycol) (PEG) electrospun membranes. Degradation in otitis-media-simulating solution was tested and it was confirmed that it was stable after 4 months. Cell viability, adhesion and collagen type I expression were confirmed using fibroblast differentiated hMSCs. When electrosprayed with a chitin solution, pro-inflammatory cytokines involved in chronic otitis media, namely IL-8 and TNF-α were found. At the same time, the innate immune response of keratinocytes was strengthened by upregulating the antimicrobial peptide HDB-2 and the anti-inflammatory cytokine TGF-β [130]. Anand and collaborators used PEOT/PBT in combination with chitin and PEG as compatibilising agent for fabrication of an electrospun membrane. Macroindentation based FEM analysis demonstrated a Young’s modulus of 37 MPa (Table 8). Cytocompatibility tests confirmed a high cell viability of cultured OC-k3 (inner ear cell line), HaCaT (keratinocyte cell line) and PC-12 (rat adrenal medulla cell line) cells and cell adhesion and proliferation was shown [131].

### 5.5. PVA in Combination with Chitosan-Based Scaffolds

Poly(vinyl alcohol) (PVA), as one of the most popular water-soluble biopolymers, is biocompatible, biodegradable, nontoxic and odourless (Figure 3e) [181]. It is generally classified into partially or fully hydrolysed which has an impact on solubility and mechanical properties [182]. As PVA was one of the first materials to be electrospun, this process is well established and attracts great attention in biomedical applications [183]. To endorse this, Pathan and collaborators have studied cytotoxicity of electrospun PVA fibres and came to the conclusion that PVA is completely noncytotoxic before electrospinning. After electrospinning, however, a cytotoxic effect toward the cells at a threshold molar concentration varying according to PVA molecular weight could be seen; increasing molecular weight resulted in increasing cytotoxicity [183]. Therefore, electrospun PVA should be treated with caution. Chitosan ((1 → 4) 2-amino-2-deoxy-β-D-Glucan) is a polysaccharide derived from chitin [184]. Table 11 gives a summary and comparison of the PVA-based scaffolds in combination with chitosan, based on mechanical properties, scaffold thickness, water contact angle, acoustic properties and porosity. In Table 12, in vitro tests in regards to TM, silk fibroin membranes are compared with adhesion and proliferation, migration, orientation and collagen analysis of the chosen cell type.

Huang and collaborators fabricated chitosan and PVA electrospun membranes, crosslinked by glutaraldehyde to counteract dissolving. The produced membrane exhibited a thickness of 18 µm, a pore size of 224.8 nm to 1380 nm, a water contact angle of 50.1 ± 3.1° (hydrophilic) and a tensile strength of 12.93 ± 0.71 MPa after 15 min of crosslinking time. For cytocompatibility tests, the authors chose human umbilical vein cells, fibroblasts and MSCs, revealing that the proposed scaffold could support mineralised tissue formation, regardless of the toxic glutaraldehyde [132]. To overcome the problem of toxic crosslinking, Chen and collaborators tested crosslinking of electrospun chitosan/PVA membranes with maleic acid, tartaric acid, citric acid and malic acid. The group found that the uncrosslinked membrane displayed average tensile strength of 3.97 ± 0.23 MPa and an average tensile modulus of 11.84 ± 0.45 MPa, while the membrane crosslinked with maleic acid showed the best suitable mechanical properties when compared to the human TM. Cell adhesion and proliferation were tested with human umbilical vein endothelial cells and human skin fibroblasts, with best results on the membranes crosslinked with maleic acid and citric acid. Chen and collaborators came to the conclusion that the chitosan/PVA/maleic acid membrane showed the best suitable properties for a TM scaffold [133].

### 5.6. PCL Based Scaffolds

Poly(ε-caprolactone) (PCL) is a semicrystalline aliphatic linear polyester (Figure 3h) and is used in the United States Food and Drug Administration (FDA)-approved surgical implants and drug delivery devices for regenerative medicine applications and tissue engineering [185,186]. As this widespread use is due to its favourable attributes such as biocompatibility, biodegradability, elasticity, availability and cost efficacy [187]. If electrospun, the obtained PCL fibres have a smooth surface, without any beaded effects. Membrane porosity ranges from 70 to 90% (*v*/*v*) and a mean value of 3.8 MPa was determined as Young’s modulus [188]. PCL electrospun membranes are inherently hydrophobic, resulting in poor wettability, of limited cell attachment and uncontrolled biological interactions with the surrounding tissue. To circumvent this disadvantage, PCL is blended with biologically active components, such as collagen [189], silk fibroin [190] or gelatine [191], which provide sufficient cues for cellular interactions [113]. Hydrophobicity is also the cause of its slow degradation rate of two to four years in bulk. Dias and collaborators however, analysed electrospun PCL membranes degradation after 90 days both, in vivo and in vitro, and demonstrated that degradation was evident in both cases, making PCL electrospun membranes applicable for skin tissue applications [187]. Table 13 gives a summary and comparison of the PCL-based scaffolds, based on mechanical properties, scaffold thickness, water contact angle, acoustic properties and porosity. In Table 14, in vitro tests in regards to TM scaffolding for PCL-based scaffolds, including PCL/silk fibroin-based scaffolds are compared with adhesion and proliferation, migration, orientation and collagen analysis of the chosen cell type.

#### 5.6.1. Additive Manufacturing of PCL-Based Scaffolds

Kozin and collaborators designed and fabricated 3D-printed PCL-based TM scaffolds, infilling the printed skeleton structure with a fibrin-collagen hydrogel mimicking the extracellular matrix. The design exhibited desirable properties when compared to human temporalis fascia, especially when designed with a higher filament count affecting the mechanical load favourably. Acoustical analysis showed that the printed scaffolds could reproduce TM motion and vibrational behaviour across different frequencies. When compared to PLA-based scaffolds, PCL-based scaffolds showed higher load-bearing capacities [100]. Based on this study, Rostam-Alilou carried out vibro-acoustic analysis of the proposed PCL-scaffolds in silico (Table 8). PCL-based scaffolds showed displacement and velocity patterns under sound in satisfying correlation to those of the native TM; the overall acoustic behaviour was not dependent on the different filament count [125]. Liu and collaborators used a combinatorial approach utilising 3D-printing assisted electrospinning of PCL and gelatine to generate mechanically tunable and vascular supportive nanofibrous membranes. The fabricated membranes showed tensile strengths of 9.8 ± 0.4 MPa, values decreasing with higher gelatine content. Biocompatibility was evaluated using HUVECs (Zhongqiaoxinzhou Biotech Co., Ltd., China), showing high viability and vascular cell responsiveness [126].

#### 5.6.2. Melt Electrowriting of PCL-Based Scaffolds

**Figure 5:**
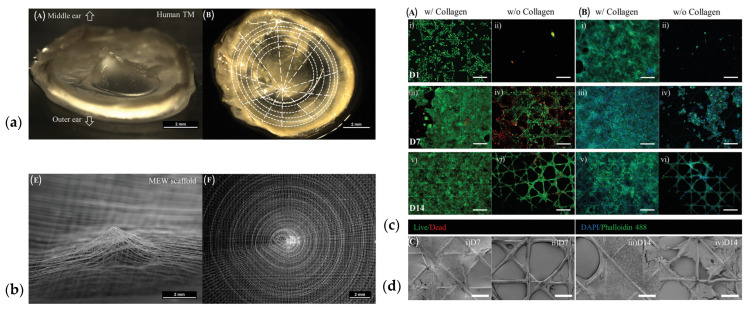
(**a**) Stereo microscopic image of a human tympanic membrane with (A) a collapsed curvature towards the middle ear and (B) as top view. The additional white lines indicate the assumed radial and circumferential arrangement of the collagen fibres. (**b**) The melt electrowritten, porous PCL scaffold mimicking the 3D curvature of the tympanic membrane and the circumferential and radial collagen fibre orientation. (**c**) Viability, adhesion and proliferation of human keratinocytes grown on the prepared melt electrowritten scaffolds with or without collagen coating for 14 days. (A) Live dead staining; viable cells appear in green, dead cells in red. (B) DAPI/Phalloidin staining (DAPI for nuclei in blue, Phalloidin for actin in green) demonstrating morphology and density of the cell layers. D1: after 1 day, D7: after 7 days, D14: after 14 days. (i), (iii), (v) with collagen; (ii), (iv), (vi) without collagen (**d**) SEM images show the typical, polygonal morphology of keratinocytes, 3 kV, magnification 200×, 5 mm, Scalebar indicates 100 µm. (Adapted from [123], licensed under CC BY 2.0).

Von Witzleben and collaborators applied melt electrowriting of PCL to create multiple designs biomimicking the human TM (Figure 5a,b; the influence of the design parameters (thickness, fibre diameter, layer to layer orientation and fibre spacing) were investigated, showing a strong influence of the scaffold thickness and the layer orientation on the bending stiffness. As the scaffold with the best properties, a four layered, with 45°—layer orientation, a fibre diameter of 10 µm and a fibre spacing of 250 µm was found, showing the best compliance in the vibrational measurements when compared to the native TM. When sealed with collagen, the scaffold supported the formation of a neo-epithelial layer faster (Figure 5c,d) [123]. Based on these results, the same authors printed PCL rings using FDM, then gel plotted a sacrificial pyramid to establish the funnel shaped form of the scaffold and subsequently melt electrowrote the PCL fibres of the scaffold. The created meshes were additionally coated in collagen to create air-tight membranes. The inclusion of radial and circumferential fibre structures resulted in good mechanical properties, and a close resemblance to the acoustic properties of the TM. The collagen coating led to a strong increase in cell number and simulation of in vivo ingrowth behaviour of HEKn and NHDFs displayed sufficient cell growth and oriented cell growth of the original collagen fibre structure of the TM [135].

#### 5.6.3. Electrospinning of PCL and PCL/Silk Fibroin-Based Scaffolds

**Figure 6:**
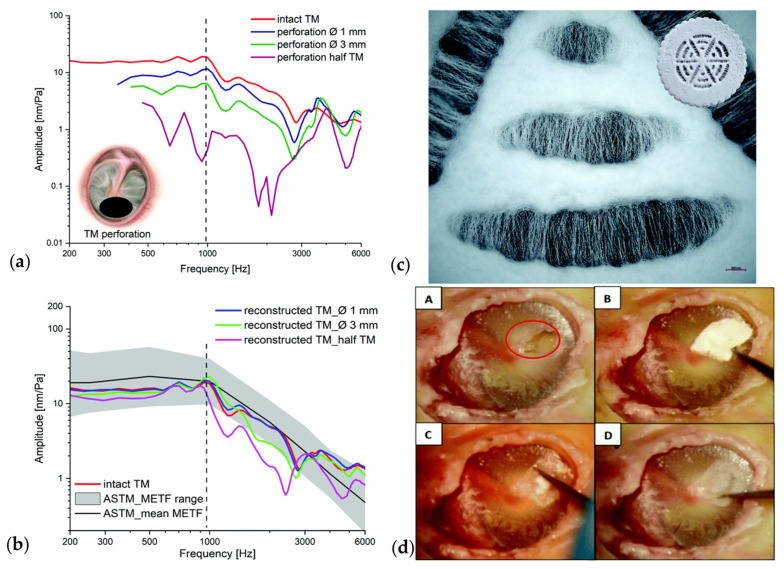
(**a**) Middle ear transfer function (METF) curves measured at the middle point of the temporal bone with intact TM and perforations of varying sizes. (**b**) METF curves of a reconstructed tympanic membranes with SF:PCL membranes. (**c**) Light microscopy image of the radial and circumferential fibres in the tympanic membrane scaffold (scale bar 200 µm). In the top right corner the whole design is shown (∅ 10 mm). (**d**) Handling test by experienced surgeons in human temporal bone: (**A**) human tympanic membrane with perforation (red); (**B**) dry SF:PCL membrane; (**C**) moistening of the membrane; (**D**) closed tympanic membrane perforation. (Reproduced from [61] under a CC-BY 3.0 licence).

Milazzo and collaborators investigated PCL electrospun membranes, yielding smooth and homogenous fibrous meshes. Young’s modulus was determined to be at 5.3 ± 0.8 MPa. FE analysis in silico showed a close mimicking of the native tissue, as per the acousto-mechanic characterisation (Table 8, Figure 6a,b) [127]. Chen and collaborators systematically investigated the effect of fibre and filament arrangement on the acousto-mechanical behaviour of electrospun PCL scaffolds, also reinforced with precrystalised PCL-filaments, by FE analysis (Table 8). Results showed that centred circular fibres at the umbo contributed immensely to a better acoustic vibrational behaviour and radial fibres provided better mechanical stability. Best results were achieved for a fraction of both fibres at 1:1. It was shown that a conical shape is essential for a successful TM reconstruction [174]. In combination with graphene oxide, Milazzo and collaborators developed electrospun scaffolds as well. In silico, using FEM analysis, the prepared scaffold showed a sharp anti-resonance, with 9 dB lower in amplitude than the mean experimental curve; the addition of graphene oxide did not lead to significant changes [127].

To overcome the wettability issues of pure electrospun PCL, Lee and collaborators fabricated electrospun scaffolds using PCL and silk fibroin, with water contact angles of 73 ± 1.2°, indicating improved cell attachment properties. Proliferation and cell viability was confirmed using human dermal fibroblasts [134]. Benecke and collaborators produced silk fibroin PCL electrospun scaffolds with radially and circumferentially oriented fibres (Figure 6c) by specific manipulation of the electrical field during the spinning process and investigated the relationships between stiffness, thickness and mass, fibre structure at the microscale, including diameter, orientation and porosity, showing that the oscillatory and mechanical properties of their TM scaffold could be readily tuned by adjusting the specific parameters. Aligned fibres lead to a 3-fold increase in Young’s modulus when compared to randomised fibres; higher spinning voltage, larger spinning distance and shorter spinning time lead to significantly better vibrational behaviour, showing comparable acousto-mechanical properties to the native human TM (Figure 6a,b. The proposed scaffolds were validated by implantation into cadaveric temporal bones; the scaffolds were free tailorable, demonstrating good handling qualities and transparency (Figure 6c). In the explants, the natural vibrational could be recovered for perforations as large as 3 mm (Table 3). Biocompatibility was confirmed using primary human keratinocytes and bronchial epithelial cells, displaying a cell viability of over 80% [61].

## 6. Conclusions and Future Perspectives

Tympanic membrane perforations frequently occur and while most perforations heal without interfering, as soon as the perforation reaches a chronic nature, intervention becomes necessary. While the gold standard has been tympanoplasty using autologous grafts, these come with various limitations, such as possible occurring donor site morbidity, a lack of transparency for future check-ups, and a lack in quality in biomechanical and acousto-mechanical material properties when compared with the native TM [49]. Success rate is only about 80% and rejection or graft displacement may require another surgery [192]. Therefore, different materials have been proposed as scaffold material. In the previous sections, fibre-based scaffolds made from different materials were presented. Figure 4 provides a flow chart detailing a schematic overview over the clinical problems addressed by tympanic membrane scaffolds, functional requirements, types of scaffolds and corresponding materials and scaffold evaluation. Different scaffold materials come with different material properties such as biocompatibility and degradation, which are summarised in Table 15.

Fibre-based membranes have been researched to the greatest extent to the authors knowledge, offering several clinical studies for the use of bacterial cellulose and silk fibroin membranes [154,155,156,157,158,159]. Due to the nature of its preparation process, the prepared membranes exclude the possibility of biomimicking the collagen structure of the native TM, but offer great biocompatibility, ease of handling during surgery and transparency for post-surgery examinations.

This drawback is easily overcome by additive manufacturing, namely 3D-printing, which offers great adaptability and flexibility concerning the proposed design and its translation into reality. This is especially relevant concerning the intricate structure of the collagen fibres of the TM, which can be easily manufactured using 3D-printing, with great detail and a easily variable number of fibres [100]. However, the minimal feature size of 3D-printing is limited, exceeding the natural thickness of a native TM and especially the pars tensa, where most perforations occur [88,100]. While offering similar acoustic properties, 3D-printed scaffolds exhibited with thicknesses ranging from 322 [88] to 616 µm [100], exceeding the natural thickness of a native TM. Only Ilhan and collaborators produced 3D-printed scaffolds with a thickness of 80 µm, but did however not test the acoustic response [124]. To counteract this, von Witzleben used melt electrowriting to produce scaffolds and successfully produced a biomimetic scaffold, mimicking the fibrous collagen structure [123,135] and the funnel shaped structure of the native TM [135], while demonstrating close to natural vibrational and acousto-mechanic behaviour of the scaffold and in vitro cellular oriented growth along the structure of the scaffold and collagen expression.

The inherent larger pore size of additive manufacturing processes leads to the need to further seal the scaffolds to prevent leakage and the invasion of pathogens into the middle ear. Mostly, collagen hydrogel is the chosen solution [123,135], but gelatine hydrogel has also been used [126], also creating the need for additional crosslinking, since the hydrogel would degrade otherwise easily [94]. Another approach to overcome the large gaps in additive manufacturing processes is the combinatorial approach of 3D-printing and electrospinning [88,126]. In the case of Mota and collaborators, the combinatorial approach yielded similar acoustic properties for the proposed scaffolds in silico, although with a much higher thickness than the native TM [63,88]. For keratinocyte adhesion it has been found that a randomised fibres limit migration of keratinocytes, as opposed to a scaffold with aligned fibres, which was shown to guide cell growth in vitro [201], which can be achieved using additive manufacturing and electrospinning techniques. Fibre-based scaffolds fabricated using electrospinning technology, offer extensive extracellular matrix alike structures, while also offering the possibility of establishing the collagen-like network in the scaffold, further supporting cellular growth [61,202]. However, electrospinning still relies on mostly toxic solvents, such as HFIP [61].

**Figure 7:**
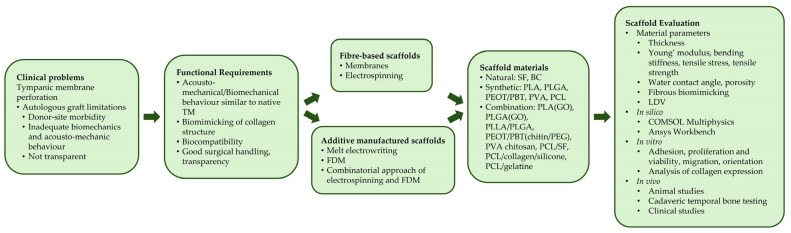
Flow chart addressing the clinical problems associated with TM perforations, functional requirements for TM scaffolds, fabrication methods and corresponding possible materials and subsequent scaffold evaluation.

The different materials proposed as TM scaffolds and their respective performance as TM scaffolds as given in Figure 7, either in additive manufacturing processes, electrospinning, or fibre-based membranes, are summarised in more detail in Table 16. Membranes prepared from bacterial cellulose and silk fibroin offer the advantage of being prepared directly from biological polymers, excluding the need for synthetic polymers. The proposed PLGA and PLLA based scaffolds have been shown to be stiffer than the native TM. A combination of both has been tested in vivo, however, hearing gain was not evaluated, which remains a crucial factor for TM replacements. When added graphene oxide, PLA showed similar properties to the native TM, but as graphene oxide is not degraded but retains in different organs and the proposed scaffolds with graphene oxide were not evaluated in vitro, this should be evaluated further. The scaffolds proposed using PVA and chitosan mostly yield much stiffer scaffolds than the native TM, and is possibly reliant on toxic crosslinkers. Green-crosslinked scaffolds were evaluated in vitro, but not in vivo.

PLA-based scaffolds are biocompatible and show a moderate degradation rate behaviour. The studies show that the proposed scaffolds show a sufficient Young’s modulus. However, PLA is hydrophobic, thus possibly hindering cellular adhesion to the scaffold. In vitro studies are lacking and should be researched further. PEOT/PBT based scaffolds were evaluated thoroughly, and were produced biomimicking the native collagen structure of the TM using additive manufacturing in combination with electrospinning. Even though scaffolds were more flexible than the native TM, FE simulations showed similar acousto-mechanical properties to the native TM and in vitro studies confirmed good cellular adhesion and proliferation of different cell types. This, however was not confirmed in in vivo studies. For PCL-based scaffolds FEM-analysis showed that PCL-based scaffolds biomimicking the native collagen structure have similar acousto-mechanical properties to the native TM. Conversely, scaffolds were found to by hydrophobic, possible interfering with cellular adhesion, but this was not confirmed in vitro. Using melt electrowriting, PCL scaffolds can be fabricated to closely mimic the inherent collagen and funnel shaped structure of the native TM, while also demonstrating similar acousto-mechanical properties. In contrast, the mixture of PCL and silk fibroin offers a close to natural acousto-mechanical-properties when electrospun, as demonstrated in cadaveric temporal bones, cytocompatibility and can be fabricated biomimicking the inherent collagen structure of the TM [61]. However, in vivo studies are still lacking.

The structure of the TM scaffold greatly influences the activity in regards to the acousto-mechanic performance and sound conduction later on. Designing the scaffold with attention to the anisotropic structure of the collagen fibres and the inherent cone shape of the native TM remains imperative. It has been shown that the acousto-mechanical properties of the native TM greatly depend on the inherent collagen-fibre arrangement, which a synthetic scaffold should take into account. In addition, Kozin and collaborators demonstrated experimentally, that the number of fibres does not significantly influences scaffold performance [100], which was later verified in silico by [125]. Using MEW, von Witzleben and collaborators have shown that additional interconnection of the and different fibre spacing allows further adjustment of the scaffold properties [135]. During electrospinning, Benecke and collaborators showed that aligned fibres lead to a higher Young’s modulus using a PCL silk fibroin mixture [61]. Using a combinatorial approach of electrospinning and FDM, Anand and collaborators suggested a geometrical dependency on the scaffold design, with the radial fibres exhibiting greater influence on the acoustic properties, but with a higher thickness than the native TM [63]. The conical structure of the TM has also been suggested to play an important role in sound conduction to the cochlea [4,62]. Von Witzleben and collaborators fabricated a conical TM scaffold using MEW demonstrating similar mechanical and vibrational properties to the human TM [135]. Benecke and collaborators manufactured a TM scaffold using electrospinning in a conical shape, showing similar mechanical and acousto-mechanical properties to the native TM [61]. Milazzo and collaborators demonstrated that a conical shape is essential for a successful TM reconstruction [127]. Taken together, it is suggested that a fibrous structure similar to the collagen structure of the native TM and a conical shape positively influence acousto-mechanical performance of a TM scaffold (Table 16).

While in vitro studies offer valuable insights into cytocompatibility and cellular behaviour on the scaffold, as well as offer more easily accessible to further investigations of collagen deposition on the scaffold and elucidation of the cellular migratory and proliferation behaviour, in vivo studies remain inevitable for biomedical applications. In vitro cellular responses do not translate directly into the complex metabolic and physiological occurrences observed in living animals and do not account for possible inflammatory or immune responses and do not reflect upon long-term behaviour, degradability or functional integration. Finally, a great support for the research on mechanical properties and acousto-mechanic behaviour, is FE simulation; with FE simulation, in a close to natural setting, the acousto-mechanical properties of the proposed scaffold can be researched, without in vivo or clinical studies, but still are limited to calculating power and limitations of the applied model. As such, in vivo studies still remain a vital part for biomedical scaffold research. After this, the next inevitable step remains clinical studies, as in vivo studies, due to species inherent differences do not directly translate to human beings. But before clinical studies can be done, further research for tympanic membrane scaffolds needs to be done, focussing on the following:

- Optimising the acousto-mechanical properties of the scaffolds, by adjusting material composition and modification of the fibrous structure to mimic the inherent collagen structure of the native collagen-fibre arrangement.
- Elucidating the metabolic, inflammatory and immune responses physiological effects in vitro and in vivo, also in consideration to biodegradation and metabolism of degradation products.
- Developing of scalable and continuous manufacturing for tympanic membrane scaffolds.
- Investigating of scaffolds used for drug-release of chronic tympanic membrane diseases, such as chronic otitis media.

Overall, it has been shown that fibre-based scaffolds show great potential and material diversity for tympanic membrane scaffolds, offering great tailorability in regards to the specific requirements for the tympanic membrane, considering both material compositions and acousto-mechanical properties.

## Figures and Tables

**Table 2:** Overview of silk fibroin membrane and bacterial cellulose scaffold properties. n.a.: not available.

Ref.	Scaffold Material	Mechanical Properties [MPa]	Thickness [µm]	WCA [°]	Acoustic Properties	Porosity
[136]	SFM	TS: 9.7	n.a.	45.6–52.2	n.a.	n.a.
[49]	SFM	n.a.	33	n.a.	n.a.	n.a.
[137]	BC	TS: 11.85 ± 2.43 YM: 11.90 ± 0.48	10.33 ± 0.58	31.17 ± 4.28	n.a.	n.a.

**Table 3:** Overview of in vitro results for silk fibroin membrane and bacterial cellulose scaffolds. TM: tympanic membrane; n.a.: not available.

Ref.	Scaffold Material	Cells	Adhesion, Proliferation, Viability	Migration	Orientation	Collagen Analysis
[49]	SFM	TM Keratinocytes	Good growth, proliferation, maintaining lineage	n.a.	n.a.	n.a.
[137]	BC	Rat primary TM fibroblasts and keratinocytes	Good adhesion and cell viability	enhanced	n.a.	n.a.

**Table 4:** Overview of pre-clinical test performed for different scaffold types and materials in regards to in vivo experiments and human cadaveric temporal bone testing. n.a.: not available.

Ref.	Type	Scaffold Material	Healing Time [Days]	Hearing	Healed TM
[136]	Sprague-Dawley	SFM	7.2 ± 1.48	n.a.	Orderly pattern of epithelial cells
[137]	Sprague-Dawley	BC	14	Average auditory threshold: 14.5 ± 1.5 Db (114%)	Thickness similar, 3 tissue layers, structures more irregular, denser collagen fibres
[128]	Sprague-Dawley	PLLA/PLGA	30	n.a.	Uniform, healthy tissue, regular cellular distribution
[61]	Cadaveric temporal bone	PCL silk fibroin	n.a.	Natural vibration behaviour can be fully restored for small perforations, vibration behaviour until the first resonance almost completely restored	n.a.

**Table 5:** Overview of clinical studies performed with different scaffold materials. TMP: tympanic membrane perforation. n.a.: not available.

Ref.	Perforation	Scaffold Material	Closure Time	Closure Rate	Hearing Gain	Surgery Time [min]
[154]	traumatic TMP	SFM	13.7 ± 4.7 days	92.3%	n.a.	n.a.
[155]	TMP present for 6 months	SFM	12.0 ± 9.3 weeks	70%	80.5 ± 27.2%	13.7 ± 4.96
[156]	TMP longer than 6 months	BC	n.a.	81.3% after 6 months	Average air threshold: 5.69 (range 5–75) Db Average air-bone gap: 5.63 (0–25) Db	n.a.
[157]	TMP secondary to chronic otitis media	BC	30 days	90%	n.a.	14.06 ± 5.23
[158]	Small/moderate TMP	BC	Small: 3.11 ± 0.84 weeks Moderate: 5.03 ± 0.69 weeks	Small: 100% Moderate: 85%	Post-surgery air-bone gap: 6.68 ± 0.29 Db	15.01 ± 0.46
[159]	traumatic TMP	BC	n.a.	n.a.	Speech recognition threshold: 13.13 ± 7.49 Dbhl after film placement	n.a.

**Table 6:** Overview of PLA, PLGA and PLLA scaffold properties. GO: graphene oxide. n.a.: not available.

Ref.	Scaffold Material	Mechanical Properties [MPa]	Thickness [µm]	WCA [°]	Acoustic Properties	Porosity
[127]	PLA, ES	YM: 37.7 ± 7.9 TS: 3.2 ± 0.2	50	102	in silico	91%
[127]	PLA/GO, ES	YM: 53.0 ± 11.3 TS: 4.0 ± 0.1	50	88	in silico	88%
[127]	PLGA, ES	YM: 118.0 ± 6.0 TS: 2.7 ± 0.2	50	115	in silico	87%
[127]	PLGA/GO, ES	YM: 189.0 ± 14.7 TS: 4.9 ± 0.4	50	112	in silico	86%
[88]	PLGA, ES	n.a.	26.22 ± 12.71	n.a.	n.a.	Pore size: 3–30 µm
[128]	PLLA/PLGA, ES	TSt: 1.3 ± 0.3 N YM: 0.08	30–90	79 ± 1	n.a.	n.a.
[100]	PLA, FDM	n.a.	604 ± 12	n.a.	Similar motion patterns at >1000 Hz, different at 400 Hz	n.a.
[124]	PLA, FDM	TSt: 4.72	80	n.a.	n.a.	Pore size: 160.4 µm

**Table 7:** Overview of in vitro restults of PLA, PLGA and PLLA scaffolds. hMSC: human mesenchymal stem cell, MSC: mesenchymal stem cell. n.a.: not available.

Reference	Scaffold Material	Cells	Adhesion, Proliferation, Viability	Migration	Orientation	Collagen Analysis
[88]	PLGA, ES	hMSCs	Cell viability was maintained, cells adhered but not fully stretched out	Interaction with the pattern reduced, preferential self-aggregation	Cells only occasionally located at scaffold radii	n.a.
[128]	PLLA/PLGA, ES	Fibroblasts, keratinocytes	Good cellular interaction with and scaffold, good proliferation, adhesion	Facilitated cell migration by porous structure	n.a.	n.a.
[124]	PLA, FDM	MSCs	Cell viability 217.8%, good attachment	n.a.	n.a.	n.a.

**Table 8:** Overview of finite element (FE) simulations for different scaffold materials. RF: radial fibres, CF: circumferential fibres.

Ref.	Scaffold Material	Software	Limitations	Acousto-Mechanic Properties
[63]	PEOT/PBT, combinatorial	COMSOL Multiphysics, 5.4	All domains were assumed as linearly elastic	Constant sound pressure of 0.02 Pa; an increasing trend in resonant frequency with rising stiffness. YM: 9.42 MPa (RF) YM: 6.75 MPa (CF) YM: 9.97 MPa (Both)
[174]	PCL, conically, different fibre arrangements	Ansys Workbench	Boundary conditions at the periphery were assumed to be fully clamped, individual fibre arrangement designs of the models were normalised	Amplitude at 710 Hz: RF: 37.2 µm/Pa; CF: 22.9 µm/Pa; Both: 35.7 µm/Pa Maximum dislocation at 5 kPa: RF: 589 µm; CF: 432 µm; Both: 452 µm Dislocation at umbo at 5 kPa: RF: 378 µm; CF: 396 µm; Both: 364 µm
[127]	PCL(GO), ES	COMSOL Multiphysics, 6.1	Linearised values are used and material parameters are assumed to be constant over the TM surface, except the thickness	Vibration patterns (250 Hz/ 1000 Hz/4000 Hz) Umbo velocity: reasonably within standard deviation of experimental results
[127]	PLA(GO), ES	COMSOL Multiphysics, 6.1	Linearised values are used and material parameters are assumed to be constant over the TM surface, except the thickness	Vibration patterns (250 Hz/ 1000 Hz/4000 Hz) Umbo velocity: reasonably within standard deviation of experimental results
[127]	PLGA(GO), ES	COMSOL Multiphysics, 6.1	Linearised values are used and material parameters are assumed to be constant over the TM surface, except the thickness	Vibration patterns (250 Hz/ 1000 Hz/4000 Hz) Umbo velocity: reasonably within standard deviation of experimental results
[131]	PEOT/PBT (chitin/PEG), biomimetic, different fibre arrangements	COMSOL Multiphysics, 6.0	All theoretical computations were performed within the linear elastic regime	comparable mechano-acoustical response to native TM; eigenfrequency analysis validated, identical acoustic structure interaction by similar modes of vibrations YM: 37 MPa
[125]	PLA, FDM	COMSOL Multiphysics	1.68 mm is calculated as for the maximum size of the elements of the models, the outer border of the models is restricted by boundary conditions	Satisfying correlation with the experimental measured values, especially at mid-to-high frequencies, the model resembled the native TM closely
[125]	PCL, FDM	COMSOL Multiphysics	1.68 mm is calculated as for the maximum size of the elements of the models, the outer border of the models is restricted by boundary conditions	Satisfying correlation with the experimentally measured values, well matching with the frequency range patterns of the native TM

**Table 9:** Overview of PEOT/PBT scaffold properties; n.a.: not available.

Ref.	Scaffold Material	Mechanical Properties [MPa]	Thickness [µm]	WCA [°]	Acoustic Properties	Porosity
[88]	PEOT/PBT, combinatorial	n.a.	95 ± 15 (Mesh) 352 ± 32 (Pattern)	n.a.	n.a.	0.003–300 μm pore size
[129]	PEOT/PBT, combinatorial	n.a.	220 ± 56	n.a.	n.a.	80 ± 0.8%
[130]	PEOT/PBT (chitin/PEG), ES	n.a.	n.a.	n.a.	n.a.	n.a.
[131]	PEOT/PBT (chitin/PEG), ES	YM: 68–80	100	n.a.	in silico	n.a.
[63]	PEOT/PBT, combinatorial	YM: CF: 6, RF: 9, Both: 13	n.a.	n.a.	in silico CF: 180 Hz, RF: 240 Hz, Both: 220 Hz	n.a.
[63]	PEOT/PBT, ES	YM: 4.5	8.88 ± 0.88	n.a.	Similar to native TM, first resonance frequency of 220 Hz	n.a.

**Table 11:** Overview of PVA/chitosan scaffold properties. n.a.: not available.

Ref.	Scaffold Material	Mechanical Properties [MPa]	Thickness [µm]	WCA [°]	Acoustic Properties	Porosity
[132]	PVA chitosan (glutaraldehyde), ES	TSt: 12.93 ± 0.71	18	50.1 ± 3.1	n.a.	Pore size: 224.8–1380 nm
[133]	PVA chitosan (maleic acid), ES	YM: 105.86 ± 6.59 TSt: 12.84 ± 2.18	n.a.	80.0 ± 0.5	n.a.	n.a.
[133]	PVA chitosan (tartaric acid), ES	YM: 164.62 ± 8.16 TSt: 7.85 ± 1.06	n.a.	77.8 ± 1.4	n.a.	n.a.
[133]	PVA chitosan (citric acid), ES	YM: 677.79 ± 51.32 TSt: 18.23 ± 0.97	n.a.	84.9 ± 0.3	n.a.	n.a.
[133]	PVA chitosan (malic acid), ES	YM: 617.18 ± 47.18 TSt: 25.51 ± 1.55	n.a.	87.8 ± 0.2	n.a.	n.a.

**Table 12:** Overview of in vitro results for PVA/chitosan scaffolds. HUVEC: human umbilical vein endothelial cell, HDF: human dermal fibroblast. n.a.: not available.

Ref.	Scaffold Material	Cells	Adhesion, Proliferation, Viability	Migration	Orientation	Collagen Analysis
[132]	PVA chitosan (glutaraldehyde) ES	HUVECs, fibroblasts, MSCs	Good proliferation and adhesion, although limited by crosslinking	n.a.	n.a.	n.a.
[133]	PVA chitosan (maleic acid), ES	HDFs, HUVECs	Good adhesion and proliferation	n.a.	n.a.	n.a.
[133]	PVA chitosan (tartaric acid), ES	HDFs, HUVECs	Adhesion, good proliferation	n.a.	n.a.	n.a.
[133]	PVA chitosan (citric acid), ES	HDFs, HUVECs	Good adhesion and proliferation	n.a.	n.a.	n.a.
[133]	PVA chitosan (malic acid), ES	HDFs, HUVECs	Adhesion, good proliferation	n.a.	n.a.	n.a.

**Table 13:** Overview of PCL, PCL silk fibroin, PCL collagen and PCL gelatine scaffold properties. BS: bending stiffness. n.a.: not available.

Ref.	Scaffold Material	Mechanical Properties [MPa]	Thickness [µm]	WCA [°]	Acoustic Properties	Porosity
[134]	PCL silk fibroin, ES	YM: 0.8 ± 0.1	n.a.	73 ± 1.2	n.a.	n.a.
[61]	PCL silk fibroin, ES	YM: 28.85 ± 7.02 BS: ~2.3 kPa	7.53 ± 0.22	n.a.	Similar, fibre mimicking enhanced acoustic response	n.a.
[123]	PCL collagen, MEW	BS: 0.41 ± 0.04	40	n.a.	Similar acoustic behaviour, low first resonance frequency	Fibre spacing: 242.8 ± 17.9
[135]	PCL collagen silicone, MEW	Lower bending stiffness than TM for all designs	Ring: 400 ± 50 Cone: 1.7 ± 0.3 mm Maximum: 300	n.a.	Close resonance peaks to TM	Spacing: 100–500 µm
[100]	PCL, FDM	n.a.	604 ± 12	n.a.	Similar motion patterns at >1000 Hz, different at 400 Hz, similar velocity to fascia	n.a.
[126]	PCL/gelatine, FDM	TSt: 9.8 ± 0.4	n.a.	86.5	n.a.	n.a.

**Table 14:** Overview of in vitro results for PCL silk fibroin and PCL collagen scaffolds. HEKn: human epidermal keratinocytes, neonatal. n.a.: not available.

Ref.	Scaffold Material	Cells	Adhesion, Proliferation, Viability	Migration	Orientation	Collagen Analysis
[134]	PCL silk fibroin, ES	HDFs	Adhesion and proliferation visible	n.a.	n.a.	n.a.
[61]	PCL silk fibroin, ES	Primary human keratinocytes, bronchial epithelial cells	Scaffold is biocompatible, no cytotoxic behaviour, cell viability over 80%	n.a.	n.a.	n.a.
[123]	PCL collagen, MEW	HaCaT	High number of living cells after 14 days	n.a.	n.a.	n.a.
[135]	PCL collagen silicone, MEW	hMSCs, NHDFs, HEKn	High cytocompatibility and cell growth; HEKn: slower cell growth	NHDFs: migration from borders to centre	Mimicking the fibre structure	Collagen expression increased from day 7 to 14 of all three types
[126]	PCL/gelatine, FDM	HUVECs	High viability, vascular cell responsiveness, non-toxicity	n.a.	n.a.	n.a.

**Table 10:** Overview of in vitro results of PEOT/PBT scaffolds. HDK: human dermal keratinocytes, NHDF: normal human dermal fibroblast; n.a.: not available.

Ref.	Scaffold Material	Cells	Adhesion, Proliferation, Viability	Migration	Orientation	Collagen Analysis
[88]	PEOT/PBT, combinatorial	hMSCs	Increase in cell viability, good adhesion	Good infiltration into electrospun mesh	Cells followed precisely circular and radial outlines,	n.a.
[129]	PEOT/PBT, combinatorial	hMSCs, keratinocytes	Good interaction (keratinocytes), uncommitted phenotype (hMSCs)	Good penetration (hMSCs)	n.a.	n.a.
[130]	PEOT/PBT (chitin/PEG), ES	Fibroblast-differentiated hMSCs, HDKs	Good viability, adhesion, interaction with the substrate	n.a.	n.a.	Collagen type I detected
[131]	PEOT/PBT (chitin/PEG), ES	hMSCs, OC-k3 cells, PC-12 cells, keratinocytes	Good adhesion, growth, with facilitated proliferation	n.a.	n.a.	n.a.
[63]	PEOT/PBT, combinatorial	NHDFs, hMSCs	Good adhesion and distribution	n.a.	Alignment with FDM fibres	Higher deposition around FDM fibres, following aligned cells
[63]	PEOT/PBT, ES	NHDFs, hMSCs	Lesser cell density	n.a.	Not as good alignment than with FDM	Diffuse collagen deposition

**Table 15:** Overview of biological properties of different scaffold materials.

Scaffold Material	Biocompatibility	Degradation
Silk fibroin	Yes	Moderate [152]
BC	Yes	Slow [193]
PCL	Yes	Slow [187]
PLA	Yes	Slow, produces acidic byproducts [194,195]
PLGA	Yes	Fast and adjustable, produces acidic byproducts [195,196]
PVA	Yes	Slow [197]
PEOT/PBT	Yes	Slow [176]
Chitin	Yes	Fast [198]
GO	Moderately	None, but retains in different organs [199]
Chitosan	Yes	Slow [200]

**Table 16:** Overview and summary of the different scaffold approaches for tympanic membrane scaffolding. n.a.: not available.

Scaffold Material	Approach	Fibrous Biomimicking	Mechanical Properties	Cell Viability	Hearing	Healed TM	References
SFM	Membrane	No	n.a.	High	Good	Good	[49,136,153,154,155]
BC	Membrane	No	More flexible	High	Good	Good	[137,156,157,158,159]
PLA	ES	No	Similar		Good	n.a.	[127]
PLA/GO	ES	No	Similar	n.a.	High	n.a.	[127]
PLGA	ES	Yes	Similar, but stiffer	Good	Good	n.a.	[88,127]
PLGA/GO	ES	No	Similar, but stiffer	n.a.	Good	n.a.	[127]
PLLA/PLGA	ES	No	More flexible	High	n.a.	Good	[128]
PEOT/PBT	Combinatorial	Yes	Similar, thicker	High	High	n.a.	[63,88,129]
PEOT/PBT	ES	No	More flexible	Good	Moderate	n.a.	[63]
PEOT/PBT (chitin/PEG)	ES	Yes	Stiffer	Good	n.a.	n.a.	[130,131]
PVA/chitosan	ES	No	Stiffer	Variable	n.a.	n.a.	[132,133]
PCL	ES	Only FE	Similar (FE), more flexible (exp.)	n.a.	Moderate	n.a.	[127,174]
PCL/GO	ES	No	Similar	n.a.	Moderate	n.a.	[127]
PCL/SF	ES	Yes	Similar	High	High	n.a.	[61,134,174,202]
PCL	MEW	Yes	Similar	High	High	n.a.	[123,135]
PLA	FDM	Yes	More flexible	Good	Good	n.a.	[100,124,125]
PCL	FDM	Yes	n.a.	n.a.	Good	n.a.	[100,125]
PCL/gelatine	FDM	Yes	More flexible	High	n.a.	n.a.	[126]

## Data Availability

No new data were created or analyzed in this study. Data sharing is not applicable to this article.

## Abbreviations

The following abbreviations are used in this manuscript:

TM	Tympanic membrane
PF	Pars flaccida
PT	Pars tensa
ECM	Extracellular matrix
LDV	Laser Doppler Vibrometry
FE	Finite element
BC	Bacterial cellulose
hMSC	Human mesenchymal stem cell
PCL	Poly(ε-caprolactone)
PEOT/PBT	Poly(ethylene oxide terephthalate)-poly(butylene terephthalate)
PLA	Poly(lactic acid)
PET	Polyethylene terephthalate
PVA	Poly(vinyl) alcohol
SFM	Silk fibroin membrane
NHDF	Neonatal human dermal fibroblasts
PEG	Poly(ethylene glycol)
FDM	Fused deposition modelling
PLGA	Poly(lactic-*co*-glycolic) acid
HDF	Human dermal fibroblast
HUVEC	Human umbilical vein endothelial cell
YM	Young’s modulus
GO	Graphene oxide
TS	Tensile stress
TSt	Tensile strength
BS	Bending stiffness
HDK	Human dermal keratinocyte
HFIP	Hexafluor-2-propanol
n.a.	Not available
ES	Electrospinning
RF	Radial fibres
CF	Circumferential fibres
MEW	Melt electrowriting
WCA	Water contact angle
